# The effect of different dietary canthaxanthin levels on water quality parameters, growth performance, biochemical indices, immunological responses, body composition, and metabolic profiles of oriental river prawn (*Macrobrachium nipponense*)

**DOI:** 10.1016/j.cirep.2025.200218

**Published:** 2025-03-11

**Authors:** Mohammad Ettefaghdoost, Hamid Navirian, Hossein Haghighi

**Affiliations:** Fisheries Department, Faculty of Natural Resources, University of Guilan, Sowmeh Sara, Guilan, Iran

**Keywords:** Canthaxanthin, Growth, Hemolymph, Gene expression, *Macrobrachium nipponense*

## Abstract

The current research aimed to evaluate the impacts of five levels of canthaxanthin (0, 50, 100, 150, and 200 mg/kg) on a total of 375 oriental river prawns, with a mean initial weight of 1.39 ± 0.07 g, over a period of 56 days. Upon completion of the culture period, the prawns were subjected to bioassays. Hemolymph was collected, and samples were taken from the hepatopancreas, intestine, muscle, and shell tissues for the analysis of physiological, biochemical, and immunological indices. The findings indicated that dissolved oxygen levels were notably increased with varying concentrations of canthaxanthin (*P* < 0.05), while other water quality parameters did not show significant variations (*P* > 0.05). The group receiving 150 mg/kg of canthaxanthin supplementation exhibited the highest growth indices and the lowest feed conversion ratio (FCR) (*P* < 0.05). The most of hemato-biochemical parameters exhibited a significant reduction in the groups that included canthaxanthin (*P* < 0.05), whereas the levels of high-density lipoprotein (HDL) and low-density lipoprotein (LDL) showed a notable increase when compared to the control treatment (*P* < 0.05). However, cholesterol, phosphorus, calcium, and uric acid levels remained unaffected by canthaxanthin supplementation (*P* > 0.05). The treatment group administered 150 mg/kg of canthaxanthin exhibited positive impacts on the majority of hemato-immune responses, in addition to enhancing the antioxidant status of the hepatopancreas. However, glutathione peroxidase (GPx) activity did not show significant differences across the experimental treatments (*P* > 0.05). Administering 150 and 200 mg/kg of canthaxanthin significantly enhanced total carotenoid content (TCC), digestive enzyme activity, intestinal microbiota, and carcass composition (*P* < 0.05). Gene expression related to growth, immune response, and metabolic processes showed significant increases in the canthaxanthin-fed treatments (*P* < 0.05). Additionally, groups supplemented with canthaxanthin demonstrated improved amino acid and fatty acid compositions (*P* < 0.05). In conclusion, the findings of this study suggest that incorporating 150 mg/kg of canthaxanthin into the diet of oriental river prawns positively impacts growth indices, as well as biochemical, immunological, and metabolic responses in this species.

## Highlights

• Canthaxanthin markedly increased growth and survival in *Macrobrachium nipponense.*
• Canthaxanthin at 150 mg/kg in oriental river prawn diets improves immuno-physiological responses.
• Canthaxanthin positively impacts digestive enzyme and antioxidant activities in M*. nipponense*.
• Canthaxanthin at 150 mg/kg improved the intestinal microbiota of oriental river prawn.
• Canthaxanthin enhanced total carotenoid content and metabolic responses in M*. nipponense*.

## Introduction

The enhancement of growth and immune performance is recognized as a critical issue in the aquaculture industry, with scholars suggesting that the reproductive efficiency of crustaceans is closely linked to the incorporation of supplemental nutrients [[1], [2], [3]]. The pivotal role of carotenoids in the nutrition and reproductive processes of aquatic animals has led to an increased demand for aquaculture products, as these pigments contribute to optimal growth, improved nutritional performance, enhanced immune response, increased market appeal, and greater adaptability to environmental fluctuations [4,5]. At present, carotenoids derived from natural sources are preferred in the aquaculture sector, primarily due to their availability and economic advantages in comparison to synthetic alternatives [6,7]. Among these natural carotenoid pigments, canthaxanthin, which is extracted from marine algae, mushrooms, vegetables, and certain aquatic products, has been identified as a valuable pigment [2,8]. While astaxanthin is widely recognized as one of the most significant carotenoids, canthaxanthin has demonstrated comparable efficacy in promoting growth, pigmentation, and immunity in aquatics [2,8,9]. Numerous studies have shown the significant effects of canthaxanthin on the growth, immune response, and overall health of crustaceans. Notably, Fawzy et al. [8] conducted research on whiteleg shrimp (Litopenaeus vannamei) and concluded that an increased dietary inclusion of canthaxanthin resulted in marked improvements in growth performance, non-specific immunity, and antioxidant status. These findings underscore the importance of incorporating this pigment into the diets of crustaceans to enhance their health status.

The oriental river prawn (Macrobrachium nipponense) is recognized as a significant and economically valuable species of freshwater prawn, classified within the family Palaemonidae and the genus Macrobrachium [[10], [11], [12]]. This species exhibits a growth rate that is roughly 20 percent faster and a higher survivability during larval stages compared to the giant freshwater prawn (Macrobrachium rosenbergii). Additionally, it demonstrates resilience to low temperatures during winter [10,11]. M. nipponense has been disseminated to various countries due to its numerous beneficial characteristics, including its adaptability to fluctuations in environmental temperatures, particularly lower temperatures, which contribute to elevated survival rates. Additionally, it exhibits superior growth performance in natural habitats and diverse production systems, including polyculture in rice paddies, pools, cages, semi-intensive and intensive culture. This prawn is also noted for its reproductive ease and significant economic viability, attributed to a relatively short reproductive cycle in comparison to other cultivated prawn species [[12], [13], [14], [15]]. M. nipponense is found in numerous rivers and reservoirs in the northern and western regions of Iran, and it also thrives in high populations in the Anzali Lagoon and the southern areas of the Caspian Sea [16,17]. Given its significant potential and the aforementioned characteristics, this species is considered an exceptionally promising option for reproduction and breeding in environments characterized by brackish and freshwater ecosystems [18,19].

Consequently, it is imperative to address the challenges related to the aquaculture of this prawn, especially in terms of developing specialized diets that can enhance growth under breeding conditions. A substantial number of studies have been conducted in this area concerning aquatics, including those by Jiang et al. [3], Zhang et al. [20], Fawzy et al. [21], Fang et al. [22], Cheng and Wu [1], Zhi et al. [23], and Niu et al. [24]. Given the notable characteristics associated with the aquaculture of M. nipponense, there is a pressing need for further investigation into dietary supplements to develop a specific and optimal commercial formulation for this species. Therefore, the current research aims to examine the impact of canthaxanthin as a dietary supplement on growth parameters, hemato-biochemical indices, immuno-physiological responses, metabolic changes, and the expression of genes associated with growth, immunity, and metabolism in the oriental river prawn, a species recognized for its profitability and favorable reproductive potential across diverse freshwater ecosystems.

## Materials and methods

### Ethics statement

All procedures related to handling and sampling were conducted in accordance with the animal care ethics guidelines established by the National Research Council [25].

### Prawn and feeding trial

The feeding experiment was carried out over a period of 56 days at the Fishland Aquarium™ (Rasht, Iran). The prawns, weighing between 1 and 1.5 g and measuring approximately 5 cm in length, were collected using nets (Danielson®, Model 30 × 33 with 48-Inch Handle, USA) from the Siah Darvishan River (lat 25°37′ N, long 49°30′ E, alt −15 AMSL, Iran). They were acclimatized in a 2000-liter tank (HWAY, Model PVC - HC1020, China) for a duration of two weeks, during which they received a basal diet formulated for oriental river prawns, presented in a 1 mm crumble. The composition of this diet included protein, lipid, ash, and moisture percentages of 45 %, 5 %, 14 %, and 9–10 %, respectively [26,27]. Subsequent to the acclimatization phase, the prawns were subjected to biological assessment utilizing a laboratory balance (Medi-Scale, Model EF3000, China) for weight measurements and a digital caliper (GUANGLU, Model 111N-101–10, China) for length measurements. The mean weight recorded was 1.39 ± 0.07 g, while the average total length was 5.04 ± 0.19 cm. Experimental prawns were randomly distributed among 15 aquariums (Fishland Aquarium™ Products, Model 800 mm length × 350 mm width × 450 mm depth/8 mm glass thickness, Iran), each with 25 prawns and 90 liters of de-chlorinated tap water. Continuous aeration was maintained using a (RESUN®, Model ACO-012, China). Water changes were performed daily, replacing 1/3rd of the aquarium volume before feeding and conducting a complete water replacement during the bioassay. To minimize potential stress on the prawns and its subsequent impact on the experiment's outcomes, a comprehensive monitoring of all external environmental factors was implemented. Throughout the experiment, efforts were made to eliminate environmental noise and prevent the introduction of uncontrolled light sources. A photoperiod consisting of a 12-hour light and 12-hour dark cycle (12L:12D) was established using LED lamps (RESUN®, Model Sa30Sl, China). The experimental diets were developed with the aid of software (LINGO™, Version 20.0, USA). The raw materials were processed using a grinder (Cemotec™, Model CM 290, Denmark) and subsequently passed through a 100-micron sieve (DG™, Model ASTM E11 standard, Iran) to ensure homogeneity and remove impurities. After weighing the ingredients on an electronic balance (Sencor, Model SKS4030, Czech Republic), the prepared components were combined using a laboratory blender (Waring® Laboratory Science, Model CB15T, USA) for 10 to 15 min. Deionized water (Neutron®, CAS No. 7732–18–5, Iran) was incorporated at a concentration of 30 % relative to the dry matter. The components were subsequently blended thoroughly, followed by the production of rations with an approximate size of 1 mm through the use of an extrusion technique (Mikim Technique Co., Ltd, Model DF-70, China). Canthaxanthin powder (CAROPHYLL® Red 10 %, DSM-Firmenich, Netherlands) was solubilized in a distilled water-oil emulsion at a ratio of 4:6, utilizing a magnetic stirrer (ZAG Shimi®, Model ZMS-74, Iran), and subsequently applied to the diets via spraying. After the drying process, the prepared diets were preserved in a freezer (Teifazmateb®, Model FR410-TC, Iran) at −18 °C. To protect the sensitive canthaxanthin pigment from light and other environmental fluctuations, daily rations were kept in airtight containers made of black polyethylene (DELTA™ Life Science, Model IPT015, Iran) in a laboratory refrigerator (Teifazmateb ®, Model 400-LT, Iran) at approximately 4 °C [26,27]. Prior to the feeding procedure, the experimental rations were stored in designated containers, and after feeding, they were promptly returned to refrigeration. The experimental protocols involved administering five distinct dietary formulations, each containing varying concentrations of canthaxanthin pigment: 0 (control), 50, 100, 150, and 200 mg/kg. Oriental river prawns were fed manually *ad libitum* 4 times daily (scheduled at 08:00, 12:00, 16:00, and 20:00 h) over a period of 56 days. This feeding regimen was implemented across 5 distinct treatments, each with 3 replications [27]. The amount of feed provided at each meal was meticulously quantified utilizing a mini scale (OHS Sterling, Model ST-502, USA) with a precision of 0.01 g prior to feeding the prawns [[26], [27], [28]]. The feed formulation and proximate analysis of the experimental diets utilized in this study are presented in Table 1 [29].

**Table 1:** Dietary ingredients and chemical analysis (dry matter basis) of the experimental diets utilized in the present study.

Canthaxanthin (mg/kg)					
	control	50	100	150	200
Ingredients (g/kg)					
Fish meal^a^	300	300	300	300	300
Soy meal	300	300	300	300	300
Wheat meal	70	70	70	70	70
Corn meal	70	70	70	70	70
Casein^b^	160	160	160	160	160
Vitamin premix^c^	20	20	20	20	20
Mineral premix^d^	20	20	20	20	20
Cholesterol^e^	2	2	2	2	2
Vitamin C^f^	1	1	1	1	1
Di-calcium phosphate^g^	5	5	5	5	5
Filler (CMC) premix^h^	52	51.95	51.90	51.85	51.80
Carotenoid pigment (canthaxanthin)^i^	0	0.05	0.10	0.15	0.20
Proximate composition (g/kg dry matter)					
Moisture	95.60	95.50	95.40	91.70	95.60
Crude protein	449.00	445.90	446.70	447.30	448.90
Crude lipid	48.90	50.30	47.50	48.40	47.20
Crude fiber	29.00	28.50	27.70	28.20	28.00
Ash	146.90	147.70	141.10	145.30	144.20
Nitrogen-free extract	230.60	232.10	241.60	239.10	236.10
Gross energy (kJ/g)^j^	18.17	18.26	18.23	18.02	18.28
Total carotenoids (mg/kg)	4.71	52.26	107.36	155.83	198.67

a Arad Podr™ (Aradax®, Iran).

b Quelab Laboratories Inc. (CAS No. 9000–71–9, Canada).

c Aras™ (Aquavit-I®, Iran)– Each 1000 g Vitamin premix contained; Retinol 1,200,000 IU, Cholecalciferol 400.000 IU, Menadione 800 mg, Thiamine 2500 mg, Riboflavin 4000 mg, Pyridoxine 2500 mg, Folate 1000 mg, Cobalamin 8 mg, Ascorbic acid 30,000 mg, Calcium pantothenate 10,000 mg, Niacin 35,000 mg, Biotin 150 mg.

d Science Laboratories (Qazvin, Iran) – Each 1000 g Mineral premix contained; Fe 6000 mg, Zn 10,000 mg, Se 20 mg, Co 100 mg, Cu 600 mg, Mn 5000 mg, I 600 mg, Choline chloride 6000 mg.

e Merck Group (Sigma-Aldrich®, CAS No. 57–88–5, USA).

f Aras™ (Aquavit-C®, Iran)– Each 500 g Vitamin C premix contained; Stay-C 35 %.

g ARAS TABAN Co.® (PHOSTAB™, Iran).

h Tehran Acid Chemical Co. (CAS No. 9004–32–4, Iran).

i CAROPHYLL® Red 10 %, (DSM-Firmenich, Netherlands).

j The calculation of gross energy is conducted utilizing the values of 16.7, 37.6 and 16.7 kJ/g for protein, lipid, and carbohydrate, respectively.

### Evaluation of water quality indicators

Water quality parameters, including temperature, dissolved oxygen, total water hardness, phosphate, nitrite, pH, nitrate, and ammonium, were assessed concurrently with each biometric measurement. Temperature was evaluated using a device (Testo SE & Co. KGaA, Model 108–2, Germany), while dissolved oxygen was determined with an instrument (Ezdo™, Model 7031, Taiwan). pH and other parameters were quantified using Milwaukee® equipment (USA), and total water hardness was assessed with a hardness tester (Ezdo™, Model 6031, Taiwan) [30].

### Evaluation of growth parameters

Feeding process was halted for a duration of 24 h following the trial, during which the samples from each experimental replication were counted and weighed. The hepatopancreas was excised while in the presence of ice (approximately at 4 °C to maintain a low operational temperature) and weighed individually (A&D, Model GF-1000, Japan) to determine its proportion relative to the total prawn weight, facilitating the calculation of the hepatosomatic index (HSI). Growth parameters were determined utilizing the following formulas [[31], [32], [33]]:

$$\mathrm{weight}\;\mathrm{gain}\;{\left(\mathrm{WG}\right)}_{\left(g\right)}=\mathrm{final}\;\mathrm{weight}\;\mathrm{of}\;{\mathrm{prawns}}_{\left(g\right)}-\;\mathrm{initial}\;\mathrm{weight}\;\mathrm{of}\;{\mathrm{prawns}}_{\left(g\right)}$$

$$\mathrm{weight}\;\mathrm{gain}\;{\left(\mathrm{WG}\right)}_{\left(\%\right)}=100\times \left[WG_{\left(g\right)}/\mathrm{initial}\;\mathrm{weight}\;\mathrm{of}\;{\mathrm{prawns}}_{\left(g\right)}\right]$$

$$\begin{array}{c} \mathrm{Spec}\mathrm{ific}\;\mathrm{growth}\;\mathrm{rate}\;{\left(\mathrm{SGR}\right)}_{\left(\%/\mathrm{day}\right)}=\\ 100\times \left[{\left(\ln.\mathrm{final}\;\mathrm{weight}\;\mathrm{of}\;{\mathrm{prawns}}_{\left(g\right)}-\;\ln.\mathrm{initial}\;\mathrm{weight}\;\mathrm{of}\;\mathrm{praw}\mathrm{ns}\right)}_{\left(g\right)}/\mathrm{trial}\;{\mathrm{duration}}_{\left(\mathrm{days}\right)}\right] \end{array}$$

$$\mathrm{Feed}\;\mathrm{conversion}\;\mathrm{ratio}\;\left(\mathrm{FCR}\right)=\mathrm{feed}\;{\mathrm{given}}_{\left(g\right)}/WG_{\left(g\right)}$$

$$\mathrm{Hepatosomatic}\;\mathrm{index}\;{\left(\mathrm{HSI}\right)}_{\left(\%\right)}=\left[\mathrm{hepatopancreas}\;\mathrm{weight}\;\mathrm{of}\;{\mathrm{prawns}}_{\left(g\right)}/\mathrm{weight}\;\mathrm{of}\;{\mathrm{prawns}}_{\left(g\right)}\right]$$

$$\mathrm{Survival}\;\mathrm{rate}\;{\left(\mathrm{SR}\right)}_{\left(\%\right)}=100\times \left[\mathrm{final}\;\mathrm{count}\;\mathrm{of}\;\mathrm{prawns}/\mathrm{initial}\;\mathrm{count}\;\mathrm{of}\;\mathrm{prawns}\right]$$

### Collection of hemolymph specimens

A syringe with a volume of 1 mL (Helma Teb™, Model DN-2481/26G-6 mm, Iran) was used to collect hemolymph from experimental prawns that had been previously rinsed with Alsever's solution (PAN-Biotech™, Catalogue No. P04-42500, Germany). Subsequently, the hemolymph was mixed with the Alsever's solution in the syringe, which was then gently and methodically agitated in an up-and-down motion to completely prevent coagulation and clotting of the collected samples. Some specimens were aliquoted into microtubes (BioPlus, South Korea) and combined with an equivalent volume of a 10 % neutral buffered formalin solution (Neutron®, Model NeutroFix® 10, Product Code 1.3300, Iran), subsequently being refrigerated at 4 °C for the purpose of immunological evaluation. Additional specimens were preserved at −80 °C in a deep freezer (Haier Biomedical, Model DW-86L579BPT, China) for further analysis. After thawing at room temperature (25 °C), the samples were subjected to homogenization utilizing a vortex mixer (KTG™, Model MFP-3500, Iran) for 20 to 30 s and then used for analytical procedures [[33], [34], [35]].

### Evaluation of hemolymph biochemical parameters

About 0.2 mL of hemolymph was mixed with an equal volume of Alsever's solution and centrifuged (Hermle AG, Model Z216 M, Germany) at 12,000 rpm for 15 min at 4 °C. The supernatant was carefully collected using a micropipette (DLAB Scientific Inc., Model MicroPette™ Plus, China) and subsequently placed into 1.5 mL microcentrifuge tubes. The biochemical indices assessed encompassed high-density lipoprotein (HDL), triglycerides, uric acid, glucose, urea, low-density lipoprotein (LDL), cholesterol, calcium, creatinine, and phosphorus. These measurements were conducted utilizing an automatic biochemistry analyzer (Biotecnica Instruments S.p.A., Model BT 4500, Italy) and commercial kits (Pars Azmoon Co., Iran). All units of measurement for hemolymph biochemical parameters are expressed in milligrams per deciliter [33,36,37].

### Evaluation of hemato-immune responses

Total hemocyte counts (THC) were assessed using the hemocytometer that was evaluated with a cover slip (Ravi Scientific Model Rohem®, India). A light microscope (YAXUN®, Model AK42, China) was utilized to examine hemocytes at a magnification of 40x. To measure differential hemocyte counts (DHC), which include semi-granular cells (SGC), hyaline cells (HC), and granular cells (GC), approximately 0.1 mL of hemolymph was used, along with an anticoagulant.

Following the air-drying of the smears, the samples were subjected to fixation in 70 % methanol (Columbus Chemical Industries, Inc., CAS No. 67-56-1, USA) for a duration of 10 min. Subsequently, the specimens were stained with Giemsa (Merck Millipore, CAS No. 51811-82-6, USA) for an additional 10 min. A total of 300 cells were counted and classified on each slide for this purpose [33,[38], [39], [40]].

A turbidimetric approach was utilized to evaluate the activity of lysozyme (LYZ), with *Micrococcus luteus* (Sigma-Aldrich®, ATCC No. 4698, USA) as the substrate. Absorbance measurements were taken at a wavelength of 450 nm with a microplate reader (BioTek®, Model ELX 808iu, USA). Phenoloxidase (PO) activity was assessed at 490 nm based on the conversion of dopachrome. The activities of lactate dehydrogenase (LDH), alanine aminotransferase (ALT), and aspartate aminotransferase (AST) were quantified at a wavelength of 340 nm, whereas the levels of alkaline phosphatase (AKP) were assessed at 405 nm. Additionally, the concentrations of total protein (TP), cortisol (CORT), and albumin (ALB) were determined utilizing diagnostic kits (Pars Azmoon Co., Iran) [33,37,40].

### Evaluation of antioxidant activities

To assess antioxidant activities, hepatopancreas specimens from prawns were subjected to a washing procedure using distilled water, subsequently weighed, and then combined with a 1:9 Tris-EDTA buffer solution (Glentham® Life Sciences, Product No. GX7489, UK). The samples were homogenized (IKA®-Werke GmbH, Model T25, Germany) at a temperature of 4 °C, followed by centrifugation at 15,000 rpm for a duration of 15 min. The supernatant was meticulously extracted with a micropipette, transferred into sterilized microcentrifuge tubes, and preserved at −80 °C for subsequent analysis. The evaluation of antioxidant activities, including glutathione peroxidase (GPx), malondialdehyde (MDA), total antioxidant capacity (T-AOC), superoxide dismutase (SOD), and catalase (CAT), was conducted using colorimetric methods (BioTek®, Model ELX 808iu, USA) in conjunction with commercial assay kits (TPR® Innovative, Code No. T-P-R/96, Iran), adhering to established protocols [33,41,42].

### Evaluation of digestive enzyme activity

In order to assess the activities of digestive enzymes, the feeding process was halted for a duration of 48 h to facilitate the complete evacuation of nutrients. Subsequently, the digestive tracts were collected and maintained on ice at a temperature of 4 °C to suppress enzymatic activity, and any remaining nutrients were eliminated. The samples were rinsed with ice-cold distilled water and weighed on an electronic balance (A&D, Model GF-1000, Japan). They were then combined with a buffer solution in a 1:9 ratio and transferred into 2 mL microcentrifuge tubes. Homogenization was performed for a duration of 30 s on ice, after which the samples were centrifuged at 10,000 rpm for 10 min at a temperature of 4 °C. The supernatant was carefully extracted and preserved at −80 °C for future analysis. Enzymatic activity was quantified through spectrophotometric techniques utilizing diagnostic kits (Pars Azmoon Co., Iran). Measurements were conducted at specific wavelengths: protease activity was evaluated at 450 nm, amylase at 540 nm, and lipase at 405 nm. The results were reported in international units per milligram of protein [[43], [44], [45]].

### Evaluation of intestinal microbiota

In order to assess the total bacterial count (TBC) and the presence of lactic acid bacteria (LAB) within the intestinal tracts of prawns, a 48-hour fasting period was implemented prior to sampling. The prawns were subjected to anesthesia using ice powder, subsequently rinsed with distilled water, and their exoskeletons were removed. Intestinal specimens were then collected, measured, and homogenized for a duration of 2 min at a ratio of 1:9 with normal saline (McKesson, Model 37-6270/0.9 %, USA) utilizing a homogenizer (IKA®-Werke GmbH, Model T25, Germany). The preparation of serial dilutions ranged between 10^−1^ and 10^−10^, and total volume of 100 μl from each dilution inoculated onto trypticase soy agar (TSA) and de Man, Rogosa, and Sharpe (MRS) media (Quelab Laboratories Inc., Canada) to assess TBC and LAB. The plates were incubated for 48 h at 25 °C (aerobic) and 30 °C (anaerobic). Bacterial counts were determined based on the logarithm of colony-forming units per gram (log_10_ cfu/g) [[46], [47], [48]].

### Evaluation of total carotenoid content

The total carotenoid content (TCC) in the specimens, specifically in the hepatopancreas, muscle, and exoskeleton, was assessed through spectrophotometric methods and reported in micrograms per gram of dry weight (μg/g dw). For this analysis, 1 g of homogenized samples was transferred into conical centrifuge tubes (Thermo Fisher Scientific, Model Falcon®, USA). Afterward, 10 mL of 98 % acetone (Thermo Fisher Scientific, CAS No. 67-64-1, USA) and 2 g of anhydrous sodium sulfate (Na2SO4) (KTA®, Iran) were introduced and thoroughly mixed for a duration of 10 min. The resulting mixtures were then subjected to filtration using qualitative filter papers (Shimi Pajouh Sahand®, Model G-4, Iran) and further purified with three aliquots of 10 mL acetone. This was followed by centrifugation at 3500 rpm for 10 min. The absorbance of the samples was subsequently measured utilizing a spectrophotometer (Cole-Parmer Ltd., Model Jenway 6850-UV/Vis, UK) at 450 nm [8,49].

### Evaluation of body biochemical compositions

The biochemical composition of the whole body, including ash content, crude protein, crude lipid, and moisture, was assessed according to the methodologies outlined by the AOAC [29]. Moisture content was determined by drying samples in an oven (Fan Azma Gostar™, Model FG-smart, Iran) at 105 °C until a constant weight was achieved. Crude protein was measured using the Kjeldahl method (N × 6.25) with a Kjeldahl apparatus (Bakhshi Laboratory Industries, Model V40, Iran), while crude lipid was evaluated through Soxhlet extraction (Bakhshi Laboratory Industries, Model K-G-6-500, Iran). Ash was determined using a laboratory chamber furnace (Fan Azma Gostar™, Model FM2P, Iran) at 550 °C for 8 h [31,47,50,51].

### Evaluation of amino acids and fatty acids compositions

Fatty acid analysis was conducted utilizing a modified protocol derived from the methodology established by Chen et al. [52]. A sample weighing 100 mg was combined with 3 mL of methanolic potassium hydroxide (KOH) (Sigma-Aldrich®, Model Titripur®, Catalogue No. 109351, USA) and incubated at a temperature of 72 °C for a duration of 20 min. Following this incubation, the mixture was allowed to cool, after which 3 mL of methanolic hydrogen chloride (Sigma-Aldrich®, Model LiChropur™, USA) was introduced, and the sample was again maintained at 72 °C for an additional 20 min. After a second cooling phase, 1 mL of n-hexane (Sigma-Aldrich®, Model MS SupraSolv®, USA) was added and permitted to stand for 8 h. Subsequently, the supernatant was subjected to centrifugation at 10,000 rpm for 2 min, and the fatty acid profiles were assessed utilizing gas chromatography (Agilent Technologies, Model 7890B GC, USA). Amino acid examination followed the methodology outlined by Li et al. [53]. Following the freeze-drying process, the specimen was subjected to hydrolysis using 6 mol/L hydrochloric acid (Sigma-Aldrich®, Model Titripur®, USA). The resulting hydrolyzed mixture was then processed through a series of steps, including dilution, evaporation, re-suspension, and additional dilution prior to filtration (Merck Millipore, Model Millex®-GP Filter Unit, Polyethersulfone pore size 0.22 μm / diameter 33 mm, USA). The amino acids present in the sample were quantitatively analyzed employing an amino acid analyzer (Hitachi High-Tech Corporation, Model VWR® LA8080, Japan).

### Evaluation of the expression of genes related to growth, immunity, and metabolism

Hepatopancreatic tissue from prawns was collected for gene expression analysis under sterile conditions. All equipment was sterilized using an autoclave (Kavosh Mega, Model L-100, Iran), and the prawn's head and thorax were disinfected with 70 % ethanol (KiaGene, Catalogue No. FPLT1191000, Iran). Tissue samples were excised with a scalpel and surgical forceps (HakimMed, Iran) and placed in sterile 1.5 mL tubes. The samples were then transferred to a liquid nitrogen tank (SJ cryo™, Model YDS-47-127, China) at −196 °C and stored at −86 °C until RNA extraction. For RNA extraction, a 100 mg specimen was collected and homogenized. One milliliter of the RNA extraction kit (SINACLON, RNX™-Plus, Catalogue No. EX6101, Iran) was utilized according to the manufacturer's guidelines. The integrity of the RNA was evaluated through electrophoresis (Bio-Rad, Model Sub-Cell® GT, USA) on a 1.0 % agarose gel (EURx®, Poland). Additionally, the purity and concentration of the RNA were determined by measuring the OD260/OD280 ratio with a spectrophotometer (Thermo Fisher Scientific, Model NanoDrop™ 2000c, USA). DNase I treatment was conducted following the protocol recommended by the Fermentas kit (Fermentas Life Sciences, Model DNase I, RNase free-EN0521, USA) before cDNA synthesis, which was conducted using a reverse transcriptase kit (SMOBIO®, Model ExcelRT™ series, Taiwan) and preserved at −80 °C. The National Center for Biotechnology Information (NCBI) database was used to retrieve gene sequences for M*. nipponense*, and the NCBI Primer BLAST tool was utilized to design primer sequences. Table 2 contains detailed information about the synthesized primers [54]. The Ampliqon (RealQ Plus 2x Master Mix Green, High ROX™, Denmark) was employed to evaluate gene expression in prawns via quantitative reverse transcription polymerase chain reaction (qRT-PCR) on an Applied Biosystems™ device (StepOnePlus™, VeriFlex™−96 Well, USA). The 25 μL reaction mixture included 12.5 μL of Ampliqon RealQ Plus 2x Master Mix Green, 2 μL of cDNA template, and 1 μL (10 μM) of both forward and reverse primers. The thermal cycling protocol comprised an initial pre-denaturation phase at 95 °C for a duration of 30 s, succeeded by a denaturation step at 95 °C for 30 s, an annealing phase at 60 °C for 30 s, and a total of 40 cycles were conducted. Each sample underwent analysis in triplicate, and the quantification of gene expression levels was performed utilizing the 2^−ΔΔCt^ method, with normalization against β-actin expression [24,51,55].

**Table 2:** Nucleotide sequences of the primers used for the quantitative real-time PCR analysis.

Gene	Primer sequences (5′−3′)	PCR product size (bp)	GenBank no.
*ACC*	Forward: ACATTGCACACCCCAGAGTA	185	KP690138.1
	Reverse: AACCCAATACGAGCACCTGA		
*A2M*	Forward: CGGCAGTAATGAACGTCCAG	160	MK439847.1
	Reverse: GCGAGGCTGAGAGGGATATT		
*Caspase*	Forward: TGCATGACAAGCCCAGACTA	217	KU942381.1
	Reverse: TGACCAAACTCCTGCAATGC		
*CDA1*	Forward: AAGAACGCCTTCCTGTACGA	205	MF360010.1
	Reverse: ATCCGGGCAAGTCTTCTTCA		
*Crustin*	Forward: ACCCGTTACCAGCTTCTTCA	241	OM032597.1
	Reverse: AAGGGAAACGCTGCTTTACG		
*CHSs*	Forward: GCATTGAGTGGCAGCTTCTT	168	KP710198.1
	Reverse: GGCTATCTCTCGTGTACCCC		
*CPT-1*	Forward: CGTTGCCTGTTCGTGTACAA	171	KP690136.1
	Reverse: AAGAACTGGCAGAGGGAGAC		
*EcR*	Forward: AGAACCCTCGGCAATCTCAA	178	MH459143.1
	Reverse: CCTTCCTCCTTCCTTCCTCG		
*ELOV6*	Forward: CTGCATGAACTACCTGGTGC	164	KU953779.1
	Reverse: CTTGTTTCACCTGGTACGCC		
*Lectin*	Forward: GCTGACGGACCAAGCCTATA	175	PP516428.1
	Reverse: ATTCCCGTTATGAGGCGTGA		
*LGR2*	Forward: TCCTTCCACTGGTCAGCATT	184	MT585155.1
	Reverse: CAGTGGTAGGCGTAGGAGAG		
*Lysozyme*	Forward: CGACACCGAACGCTACAAGG	118	AY257550.2
	Reverse: GGAACCACGAGACCAGCAC		
*RXR-S*	Forward: CCTCTCCCAGTGTGTCCAAT	197	KC460324.1
	Reverse: ACCTTTGCAACCCTCACAAC		
*Δ^9^-D*	Forward: GGGATGGCTCATGTACCGTA	168	KU922943.1
	Reverse: CAGTAAATGCAGGGGATGGC		
*β-actin*	Forward: GTGCCCATCTACGAGGGTTA	247	KY780298.1
	Reverse: CGTCAGGGAGCTCGTAAGAC		

Abbreviations: ACC: Acetyl-CoA carboxylase, A2M: alpha-2-macroglobulin, CDA1: cytidine deaminase 1, CHSs: chitin synthase, CPT-1: Carnitine O-palmitoyltransferase 1, EcR: ecdysteroid receptor, ELOV6: Elongation of very long chain fatty acids protein 6, LGR2: leucine-rich repeat-containing G-protein-coupled receptor 2, RXR-S: retinoid X receptor, Δ^9^-D: Delta-9 desaturase.

### Statistical analysis

Analysis of statistical data was performed utilizing one-way ANOVA with IBM SPSS software (Version 26.0, USA). The normality of the data was evaluated via the Kolmogorov-Smirnov test, and the homogeneity of variance was assessed using the Levene test. Mean values were determined employing Duncan's multiple range test (DMRT) at a 95 % confidence level. Subsequently, the data were systematically arranged into tables using Microsoft Excel 2016 (USA), and the results were presented in the format of mean ± standard deviation.

## Results

### Water quality parameters

Table 3 presents the findings related to the water quality parameters. Throughout the study, temperature, total water hardness, phosphate, nitrite, ammonium, pH, and nitrate showed no significant differences across treatments (*P* > 0.05). However, dissolved oxygen levels were significantly affected by variations in canthaxanthin pigment, with the control treatment showing lower levels than those in the canthaxanthin-supplemented groups (*P* < 0.05). The highest concentrations of dissolved oxygen were observed in the 150 and 200 mg/kg canthaxanthin treatments (*P* < 0.05).

**Table 3:** Effects of different dietary canthaxanthin levels on water quality indicators of *Macrobrachium nipponense,* after a 56-day feeding experiment. Values are expressed as mean ± SD (n = 3). Mean values in the same row with different superscript letters are significantly different (*P* < 0.05).

Parameters			Canthaxanthin (mg/kg)			One- way ANOVA		
	Control	50	100	150	200	F	d.f.	P-value (DMRT)
Temperature ( °C)	24.77 ± 0.14	24.92 ± 0.69	24.90±0.23	25.08 ± 0.47	25.84 ± 0.98	0.294	4	0.876
pH	6.75 ± 0.08	6.93 ± 0.22	6.86 ± 0.34	7.02 ± 0.27	7.05 ± 0.15	0.579	4	0.681
Dissolved oxygen (mg/L)	6.52 ± 0.14^c^	6.95 ± 0.10^b^	6.91 ± 0.07^b^	7.13 ± 0.09^a^	7.15 ± 0.04^a^	27.348	4	0.000
Ammonium (mg/L)	0.72 ± 0.02	0.70 ± 0.04	0.73 ± 0.03	0.68 ± 0.06	0.68 ± 0.01	0.976	4	0.465
Nitrite (mg/L)	0.12 ± 0.02	0.10 ± 0.01	0.12 ± 0.02	0.10 ± 0.02	0.10 ± 0.02	0.717	4	0.607
Nitrate (mg/L)	0.17 ± 0.01	0.19 ± 0.01	0.19 ± 0.02	0.18 ± 0.03	0.17 ± 0.02	1.135	4	0.396
Phosphate (mg/L)	0.02 ± 0.01	0.02 ± 0.01	0.02 ± 0.01	0.02 ± 0.01	0.01 ± 0.02	0.934	4	0.492
Total hardness (mg/L)	133.06 ± 2.78	129.79 ± 2.58	130.92 ± 2.39	130.68 ± 2.66	130.31 ± 2.75	1.222	4	0.371

### Growth performance metrics

The results of the investigation into the growth metrics of prawns subjected to varying dietary levels of canthaxanthin pigment are summarized in Table 4. The data demonstrate that an increase in canthaxanthin concentrations is associated with improved growth indices, FCR, and SR of the prawns, with statistically significant differences observed compared to the group that did not receive canthaxanthin supplementation (*P* < 0.05). The treatment group receiving 150 mg/kg of canthaxanthin exhibited the highest values for FBW, WG, PWG, and SGR, while the control group demonstrated the lowest values for these parameters (*P* < 0.05). Furthermore, the treatment with 150 mg/kg of canthaxanthin also resulted in the lowest FCR when compared to the group without canthaxanthin (*P* < 0.05). In contrast, the HSI did not show any significant variation among the experimental treatments (*P* > 0.05).

**Table 4:** Effects of different dietary canthaxanthin levels on growth performance metrics of *Macrobrachium nipponense,* after a 56-day feeding experiment. Values are expressed as mean ± SD (n = 3). Mean values in the same row with different superscript letters are significantly different (*P* < 0.05).

Parameters			Canthaxanthin (mg/kg)			One- way ANOVA		
	Control	50	100	150	200	F	d.f.	P-value (DMRT)
FBW (g)	3.83 ± 0.16^d^	4.42 ± 0.21^c^	4.80 ± 0.32^bc^	5.82 ± 0.42^a^	5.05 ± 0.31^b^	19.495	4	0.000
WG (g)	2.43 ± 0.20^c^	3.02 ± 0.25^bc^	3.40 ± 0.37^b^	4.42 ± 0.68^a^	3.65 ± 0.32^b^	13.123	4	0.001
PWG (%)	173.13 ± 58.63^c^	215.72 ± 16.42^bc^	242.14 ± 23.18^b^	314.76 ± 48.05^a^	260.71 ± 22.15^b^	13.059	4	0.001
SGR (%/day)	1.79 ± 0.08^b^	2.04 ± 0.18^ab^	2.19 ± 0.12^ab^	2.53 ± 0.14^a^	1.86 ± 0.17^b^	7.688	4	0.012
FCR	1.96 ± 0.04^a^	1.88 ± 0.03^b^	1.71 ± 0.05^c^	1.43 ± 0.07^e^	1.61 ± 0.02^d^	389.524	4	0.000
HSI (%)	4.65 ± 0.30	4.85 ± 0.38	4.72 ± 0.44	5.01 ± 0.61	4.98 ± 0.32	0.514	4	0.698
SR (%)	93.34 ± 5.66^b^	95.55 ± 3.85^ab^	100.00 ± 0.00^a^	100.00 ± 0.00^a^	100.00 ± 0.00^a^	3.051	4	0.047

Abbreviations: FBW: Final body weight; WG: Weight gain; PWG: Percent weight gain; SGR: Specific growth rate; FCR: Feed conversion ratio; HSI: Hepatosomatic index; SR: Survival rate.

### Hemato-biochemical parameters

Hemato-biochemical indices of oriental river prawns exposed to different concentrations of canthaxanthin are summarized in Table 5. The results show that urea, glucose, creatinine, triglycerides, HDL, and LDL levels were significantly influenced by the inclusion of canthaxanthin in the diet, with notable differences observed compared to the control group (*P* < 0.05). In contrast, uric acid, calcium, phosphorus, and cholesterol levels did not differ significantly (*P* > 0.05). The control treatment, which did not contain canthaxanthin, exhibited the highest levels of urea, glucose, creatinine, and triglycerides, all of which decreased significantly with increasing canthaxanthin concentrations (*P* < 0.05). Conversely, HDL and LDL levels significantly increased with higher canthaxanthin concentrations, with the control treatment displaying the lowest levels, while treatments with 150 and 200 mg/kg showed significantly elevated levels (*P* < 0.05).

**Table 5:** Effects of different dietary canthaxanthin levels on hemolymph biochemical indicators of *Macrobrachium nipponense*, after a 56-day feeding experiment. Values are expressed as mean ± SD (n = 3). Mean values in the same row with different superscript letters are significantly different (*P* < 0.05).

Parameters			Canthaxanthin (mg/kg)			One- way ANOVA		
	Control	50	100	150	200	F	d.f.	P-value (DMRT)
Urea (mg/dL)	13.51 ± 0.28^a^	12.62 ± 0.34^b^	12.57 ± 0.30^b^	12.25 ± 0.32^b^	12.31 ± 0.26^b^	19.239	4	0.000
Uric acid (mg/dL)	1.52 ± 0.46	1.50 ± 0.48	1.38 ± 0.54	1.49 ± 0.43	1.50 ± 0.52	0.581	4	0.866
Glucose (mg/dL)	37.57 ± 0.66^a^	35.44 ± 0.82^b^	34.98 ± 0.46^c^	31.21 ± 0.37^d^	35.63 ± 0.32^b^	93.723	4	0.000
Creatinine (mg/dL)	0.35 ± 0.02^a^	0.33 ± 0.01^ab^	0.31 ± 0.01^bc^	0.29 ± 0.02^c^	0.31 ± 0.02^bc^	9.198	4	0.005
Calcium (mg/dL)	63.90 ± 1.87	64.43 ± 2.39	63.50 ± 1.65	63.52 ± 1.98	65.12 ± 1.59	0.543	4	0.789
Phosphorus (mg/dL)	11.14 ± 0.58	11.27 ± 0.70	11.12 ± 0.29	11.19 ± 0.25	11.21 ± 0.36	0.079	4	0.997
Cholesterol (mg/dL)	44.05 ± 0.59	43.92 ± 0.17	43.59 ± 0.36	43.56 ± 0.11	43.75 ± 0.18	1.378	4	0.362
Triglycerides (mg/dL)	79.13 ± 0.46^a^	75.15 ± 0.26^b^	73.67 ± 0.58^c^	72.76 ± 0.35^d^	75.25 ± 0.30^b^	124.683	4	0.000
HDL (mg/dL)	12.21 ± 0.38^d^	13.33 ± 0.28^c^	14.46 ± 0.44^b^	15.29 ± 0.61^a^	13.98 ± 0.35^bc^	25.188	4	0.000
LDL (mg/dL)	5.47 ± 0.35^d^	6.08 ± 0.11^c^	6.75 ± 0.19^b^	7.39 ± 0.18^a^	7.21 ± 0.28^a^	45.969	4	0.000

Abbreviations: HDL: High-density lipoprotein; LDL: Low-density lipoprotein.

### Immune responses

The findings from the examination of immune responses in the experimental prawns are displayed in Table 6. Notably, the levels of ALB and TP exhibited a significant increase in prawns subjected to canthaxanthin treatments. Conversely, the CORT levels in prawns receiving canthaxanthin demonstrated a significant reduction compared to the control group that did not receive canthaxanthin (*P* < 0.05). The treatment contained 150 mg/kg canthaxanthin pigment resulted in the highest ALB, TP, LYZ, and PO levels, also the lowest CORT levels, significantly differing from other treatments (*P* < 0.05). Additionally, the parameters of cell-mediated immunity, specifically SGC, HC, THC, and GC in prawns treated with varying concentrations of canthaxanthin showed a noteworthy enhancement relative to the control group without canthaxanthin pigment (*P* < 0.05). The enzymatic activities of PO and LYZ in the canthaxanthin supplementation groups were elevated compared to the group without canthaxanthin (*P* < 0.05). Furthermore, the concentrations of AST, LDH, ALT, and AKP exhibited a statistically significant reduction when compared to the control group that did not receive canthaxanthin (*P* < 0.05).

**Table 6:** Effects of different dietary canthaxanthin levels on hemato-immune indices of *Macrobrachium nipponense,* after a 56-day feeding experiment. Values are expressed as mean ± SD (n = 3). Mean values in the same row with different superscript letters are significantly different (*P* < 0.05).

Parameters			Canthaxanthin (mg/kg)			One- way ANOVA		
	Control	50	100	150	200	F	d.f.	P-value (DMRT)
ALB (g/dL)	1.37 ± 0.03^d^	1.50 ± 0.02^c^	1.51 ± 0.02^bc^	1.54 ± 0.01^ab^	1.56 ± 0.02^a^	30.183	4	0.000
TP (g/dL)	4.93 ± 0.08^e^	6.58 ± 0.13^c^	7.37 ± 0.07^b^	7.82 ± 0.16^a^	5.84 ± 0.10^d^	384.860	4	0.000
CORT (ng/mL)	11.56 ± 0.30^a^	7.84 ± 0.24^cd^	8.38 ± 0.31^c^	7.58 ± 0.44^d^	9.40 ± 0.38^b^	73.368	4	0.001
LYZ (U/min/mL)	10.59 ± 0.36^e^	12.71 ± 0.21^d^	16.15 ± 0.44^b^	16.72 ± 0.14^a^	15.39 ± 0.31^c^	232.933	4	0.000
PO (U/min/mg protein)	0.58 ± 0.02^d^	0.65 ± 0.03^c^	0.68 ± 0.01^bc^	0.73 ± 0.02^ab^	0.75 ± 0.03^a^	27.215	4	0.000
THC (× 10^5^ cells/mL)	89.38 ± 3.42^d^	107.75 ± 5.91^c^	121.13 ± 6.52^b^	132.15 ± 4.10^a^	133.48 ± 8.63^a^	30.053	4	0.000
GC (× 10^5^ cells/mL)	8.90 ± 1.10^c^	14.76 ± 1.13^b^	17.24 ± 0.88^b^	21.29 ± 1.32^a^	21.84 ± 2.22^a^	44.297	4	0.000
SGC (× 10^5^ cells/mL)	32.35 ± 2.90^c^	38.29 ± 2.51^b^	45.52 ± 1.35^a^	49.05 ± 3.27^a^	49.48 ± 4.55^a^	18.520	4	0.000
HC (× 10^5^ cells/mL)	47.46 ± 1.76^c^	53.73 ± 2.09^b^	57.30 ± 1.02^ab^	60.74 ± 4.11^a^	61.18 ± 5.37^a^	8.014	4	0.002
ALT (U/L)	20.12 ± 2.01^a^	16.02 ± 1.24^b^	14.21 ± 1.13^bc^	12.31 ± 0.84^cd^	10.08 ± 0.41^d^	26.963	4	0.000
AST (U/L)	75.68 ± 1.06^a^	71.95 ± 0.90^b^	71.08 ± 0.69^b^	68.14 ± 1.36^c^	67.25 ± 0.33^c^	39.812	4	0.000
AKP (U/L)	158.31 ± 3.18^a^	153.08 ± 1.86^bc^	150.94 ± 1.62^c^	154.23 ± 1.87^abc^	155.34 ± 2.11^ab^	5.007	4	0.033
LDH (U/L)	645.40 ± 1.91^a^	627.25 ± 1.64^c^	626.57 ± 2.73^c^	626.06 ± 2.45^c^	632.13 ± 3.39^b^	34.065	4	0.000

Abbreviations: ALB: Albumin; TP: Total protein; CORT: Cortisol; LYZ: Lysozyme; PO: Phenoloxidase; THC: Total hemocyte count; GC: Granular cells; SGC: Semi-granular cells; HC: Hyaline cells; ALT: Alanine aminotransferase; AST: Aspartate aminotransferase; AKP: Alkaline phosphatase; LDH: Lactate dehydrogenase.

### Antioxidant activities

The findings related to antioxidant activities are detailed in Table 7. A significant increase in T-AOC of canthaxanthin-fed treatments when compared to the control treatment. Conversely, the levels of CAT, MDA, and SOD exhibited a statistically significant decrease in response to canthaxanthin treatment (*P* < 0.05). Treatments administered with 150 mg/kg of canthaxanthin exhibited the highest T-AOC, along with the lowest levels of MDA, SOD, and CAT activity (*P* < 0.05). It is noteworthy that the activity of GPx remained unchanged across the different concentrations of canthaxanthin administered (*P* > 0.05).

**Table 7:** Effects of different dietary canthaxanthin levels on hepatopancreatic antioxidant activities of *Macrobrachium nipponense,* after a 56-day feeding experiment. Values are expressed as mean ± SD (n = 3). Mean values in the same row with different superscript letters are significantly different (*P* < 0.05).

Parameters			Canthaxanthin (mg/kg)			One- way ANOVA		
	Control	50	100	150	200	F	d.f.	P-value (DMRT)
T-AOC (U/mg protein)	2.56 ± 0.29^c^	2.93 ± 0.23^bc^	3.32 ± 0.16^ab^	3.73 ± 0.27^a^	3.65 ± 0.20^a^	16.454	4	0.000
SOD (U/mg protein)	7.31 ± 0.40^a^	4.97 ± 0.61^b^	5.22 ± 0.30^b^	5.39 ± 0.35^b^	6.84 ± 0.41^a^	20.295	4	0.000
GPx (U/mg protein)	31.59 ± 1.20	31.48 ± 2.04	28.40 ± 1.84	28.83 ± 1.05	29.45 ± 1.78	2.672	4	0.117
CAT (U/mg protein)	13.99 ± 0.72^a^	9.88 ± 0.98^c^	7.94 ± 0.21^d^	8.80 ± 0.66^cd^	12.17 ± 0.76^b^	39.431	4	0.000
MDA (nmol/mg protein)	9.54 ± 0.25^a^	7.80 ± 0.41^b^	6.65 ± 0.44^cd^	6.43 ± 0.56^d^	7.36 ± 0.68^bc^	22.233	4	0.000

Abbreviations: T-AOC: Total antioxidant capacity; SOD: Superoxide dismutase; GPx: Glutathione peroxidase; CAT: Catalase; MDA: Malondialdehyde.

### Digestive enzymes activities

Table 8 illustrates the results of the assessment of digestive enzyme activity. The findings indicate that different concentrations of canthaxanthin significantly affected the activity of digestive enzymes, with a marked increase in the activities of amylase, protease, and lipase in prawns as the dietary levels of canthaxanthin increased (*P* < 0.05). The treatment groups that received 150 and 200 mg/kg of canthaxanthin exhibited the highest enzyme activities, while the control treatment displayed the lowest enzyme activities (*P* < 0.05).

**Table 8:** Effects of different dietary canthaxanthin levels on digestive enzyme activities of Macrobrachium nipponense, after a 56-day feeding experiment. Values are expressed as mean ± SD (n = 3). Mean values in the same row with different superscript letters are significantly different (*P* < 0.05).

Parameters			Canthaxanthin (mg/kg)			One- way ANOVA		
	Control	50	100	150	200	F	d.f.	P-value (DMRT)
Protease (U/mg protein)	1.41 ± 0.05^d^	1.54 ± 0.02^c^	1.60 ± 0.03^bc^	1.65 ± 0.02^ab^	1.68 ± 0.07^a^	25.594	4	0.000
Lipase (U/mg protein)	0.75 ± 0.04^c^	0.84 ± 0.02^b^	0.87 ± 0.01^b^	0.92 ± 0.03^a^	0.95 ± 0.02^a^	24.975	4	0.000
Amylase (U/mg protein)	1.85 ± 0.13^d^	2.24 ± 0.15^c^	2.45 ± 0.09^b^	2.73 ± 0.10^a^	2.62 ± 0.07^ab^	40.946	4	0.000

### Intestinal microbiota

The results derived from the analysis of intestinal microbiota are presented in Table 9. The findings reveal that TBC and LAB exhibited statistically significant differences among the various treatments (*P* < 0.05). The TBC exhibited significant reduction with increasing dietary inclusion of canthaxanthin. Specifically, treatments incorporating 150 and 200 mg/kg of the pigment resulted in the lowest bacterial counts (*P* < 0.05). Conversely, an increase in dietary levels of canthaxanthin was associated with a significant rise in LAB, with the 150 mg/kg treatment demonstrating the highest population of these bacteria in the intestines of the prawns, showing a statistically significant difference compared to the other groups (*P* < 0.05).

**Table 9:** Effects of different dietary canthaxanthin levels on intestinal microbiota of *Macrobrachium nipponense,* after a 56-day feeding experiment. Values are expressed as mean ± SD (n = 3). Mean values in the same row with different superscript letters are significantly different (*P* < 0.05).

Parameters			Canthaxanthin (mg/kg)			One- way ANOVA		
	Control	50	100	150	200	F	d.f.	P-value (DMRT)
TBC (log_10_ CFU/g)	7.54 ± 0.18^a^	7.40 ± 0.13^ab^	7.18 ± 0.21^bc^	6.93 ± 0.15^cd^	6.75 ± 0.10^d^	18.162	4	0.002
LAB (log_10_ CFU/g)	1.24 ± 0.02^c^	1.25 ± 0.05^bc^	1.28 ± 0.04^abc^	1.32 ± 0.06^a^	1.30 ± 0.04^ab^	3.829	4	0.043

Abbreviations: TBC: Total bacteria count; LAB: Lactic acid bacteria.

### Total carotenoid content

Table 10 shows the results of the TCC in different body parts of the experimental prawns. The treatment group administered a dietary supplementation of 150 mg/kg canthaxanthin exhibited the highest levels of carotenoids in the hepatopancreas, muscle, and exoskeletal tissues. Conversely, the control treatment exhibited the lowest levels of carotenoids (*P* < 0.05).

**Table 10:** Effects of different dietary canthaxanthin levels on total carotenoid content of *Macrobrachium nipponense,* after a 56-day feeding experiment. Values are expressed as mean ± SD (n = 3). Mean values in the same row with different superscript letters are significantly different (*P* < 0.05).

Parameters			Canthaxanthin (mg/kg)			One- way ANOVA		
	Control	50	100	150	200	F	d.f.	P-value (DMRT)
Muscle (μg/g)	1.44 ± 0.17^d^	17.79 ± 1.89^c^	23.35 ± 1.74^b^	25.92 ± 1.32^a^	23.61 ± 0.95^a^	188.667	4	0.000
Exoskeleton (μg/g)	3.91 ± 0.70^e^	36.59 ± 2.40^d^	67.14 ± 2.68^c^	78.88 ± 3.62^a^	72.35 ± 1.92^b^	589.393	4	0.000
Hepatopancreas (μg/g)	3.80 ± 0.87^e^	20.24 ± 1.59^d^	27.22 ± 2.41^c^	36.52 ± 2.31^b^	43.77 ± 3.29^a^	146.137	4	0.000

### Body compositions

Table 11 delineates the findings from the analysis of the body approximate analysis of the prawns. The data indicate that the moisture content in the canthaxanthin treatment groups exhibited a significant reduction, while the levels of crude lipid and crude protein demonstrated a notable increase in comparison to the non-supplemented canthaxanthin treatment (*P* < 0.05). Conversely, the assessment of body ash content indicated that there were no statistically significant differences among the experimental treatments (*P* > 0.05).

**Table 11:** Effects of different dietary canthaxanthin levels on whole-body proximate composition of *Macrobrachium nipponense,* after a 56-day feeding experiment. Values are expressed as mean ± SD (n = 3). Mean values in the same row with different superscript letters are significantly different (*P* < 0.05).

Parameters			Canthaxanthin (mg/kg)			One- way ANOVA		
	Control	50	100	150	200	F	d.f.	P-value (DMRT)
Moisture (%)	75.72 ± 0.35^a^	73.69 ± 0.41^b^	73.34 ± 0.10^b^	71.57 ± 0.23^c^	71.99 ± 0.20^c^	68.532	4	0.000
Crude protein (%)	15.86 ± 0.13^d^	16.30 ± 0.24^c^	16.85 ± 0.18^b^	18.01 ± 0.38^a^	17.69 ± 0.25^a^	43.439	4	0.000
Crude lipid (%)	2.98 ± 0.19^c^	3.77 ± 0.32^b^	4.29 ± 0.11^a^	4.33 ± 0.09^a^	4.29 ± 0.57^a^	33.487	4	0.000
Ash (%)	6.07 ± 0.31	5.95 ± 0.13	5.88 ± 0.11	5.90 ± 0.08	5.88 ± 0.06	0.069	4	0.997

### Amino acid and fatty acid composition

Table 12 presents the evaluations of body amino acid profiles, whereas Table 13 illustrates the whole-body fatty acid compositions. Prawns administered canthaxanthin exhibited notable enhancements in both profiles. An increased dietary intake of canthaxanthin resulted in substantial rises in polyunsaturated fatty acids (PUFA), monounsaturated fatty acids (MUFA), and essential amino acids (EAA), with all changes being statistically significant in comparison to the control treatment (*P* < 0.05). Conversely, elevated concentrations of canthaxanthin were linked to reduced levels of saturated fatty acids (SFA) and specific non-essential amino acids (NEAA) in comparison to the control group (*P* < 0.05).

**Table 12:** Effects of different dietary canthaxanthin levels on whole-body amino acid profiles (% dry matter) of *Macrobrachium nipponense,* after a 56-day feeding experiment. Values are expressed as mean ± SD (n = 3). Mean values in the same row with different superscript letters are significantly different (*P* < 0.05).

Amino acid			Canthaxanthin (mg/kg)			One- way ANOVA		
	Control	50	100	150	200	F	d.f.	P-value (DMRT)
EAA								
Arginine	7.52 ± 0.04^c^	7.67 ± 0.10^bc^	7.80 ± 0.13^b^	7.96 ± 0.06^a^	8.08 ± 0.12^a^	21.126	4	0.000
Histidine	2.29 ± 0.02^d^	2.36 ± 0.05^cd^	2.44 ± 0.04^bc^	2.48 ± 0.08^b^	2.59 ± 0.05^a^	11.997	4	0.001
Isoleucine	3.66 ± 0.06^c^	3.71 ± 0.03^c^	3.86 ± 0.04^b^	3.99 ± 0.05^a^	3.96 ± 0.03^a^	36.520	4	0.000
Leucine	6.06 ± 0.10^b^	6.13 ± 0.06^b^	6.38 ± 0.12^a^	6.32 ± 0.16^a^	6.46 ± 0.05^a^	9.841	4	0.002
Lysine	4.80 ± 0.10^c^	5.08 ± 0.03^b^	5.11 ± 0.05^b^	5.19 ± 0.04^ab^	5.27 ± 0.07^a^	18.673	4	0.000
Methionine	2.19 ± 0.03^c^	1.99 ± 0.02^d^	2.26 ± 0.07^bc^	2.35 ± 0.05^a^	2.32 ± 0.02^ab^	34.119	4	0.000
Phenylalanine	3.11 ± 0.06^c^	3.19 ± 0.03^bc^	3.24 ± 0.04^b^	3.30 ± 0.09^ab^	3.39 ± 0.05^a^	7.625	4	0.004
Threonine	1.99 ± 0.03^c^	2.14 ± 0.08^b^	2.26 ± 0.05^a^	2.22 ± 0.04^ab^	2.33 ± 0.05^a^	11.876	4	0.001
Tryptophan	0.80 ± 0.02^c^	0.84 ± 0.02^b^	0.87 ± 0.01^b^	0.92 ± 0.03^a^	0.95 ± 0.03^a^	25.327	4	0.000
Valine	3.62 ± 0.08^d^	3.84 ± 0.06^c^	4.00 ± 0.07^b^	4.16 ± 0.03^a^	4.24 ± 0.06^a^	58.582	4	0.000
ΣEAA	36.05 ± 0.78^c^	36.95 ± 0.66^c^	38.22 ± 0.34^b^	38.89 ± 0.57^ab^	39.77 ± 0.62^a^	16.147	4	0.000
NEAA								
Alanine	4.47 ± 0.06^b^	4.55 ± 0.04^b^	4.60 ± 0.10^b^	4.74 ± 0.04^a^	4.80 ± 0.07^a^	10.544	4	0.002
Aspartic acid	8.28 ± 0.12	8.04 ± 0.11	8.06 ± 0.07	8.00 ± 0.17	7.96 ± 0.13	3.493	4	0.067
Cysteine	1.74 ± 0.05	1.64 ± 0.02	1.70 ± 0.05	1.72 ± 0.07	1.71 ± 0.03	2.315	4	0.108
Glutamic acid	11.32 ± 0.08	11.45 ± 0.15	11.28 ± 0.23	11.38 ± 0.11	11.30 ± 0.20	0.387	4	0.789
Glycine	5.84 ± 0.11^a^	5.65 ± 0.04^b^	5.59 ± 0.07^b^	5.57 ± 0.09^b^	5.53 ± 0.12^b^	6.129	4	0.011
Proline	3.09 ± 0.06^a^	2.99 ± 0.05^a^	3.05 ± 0.09^a^	2.87 ± 0.05^b^	2.81 ± 0.04^b^	11.883	4	0.001
Serine	3.63 ± 0.04	3.65 ± 0.05	3.61 ± 0.03	3.68 ± 0.03	3.64 ± 0.02	1.636	4	0.198
Tyrosine	3.24 ± 0.06^a^	3.20 ± 0.07^a^	3.15 ± 0.05^ab^	3.03 ± 0.04^c^	3.07 ± 0.03^bc^	10.517	4	0.002
ΣNEAA	41.61 ± 0.83	41.17 ± 0.91	41.04 ± 0.49	40.98 ± 1.04	40.82 ± 0.75	0.421	4	0.796
ΣEAA/ΣNEAA	0.87 ± 0.01^d^	0.90 ± 0.01^c^	0.93 ± 0.01^b^	0.95 ± 0.01^ab^	0.97 ± 0.02^a^	28.743	4	0.000

Abbreviations: EAA: Essential amino acids, NEAA: Non-essential amino acids.

**Table 13:** Effects of different dietary canthaxanthin levels on whole-body fatty acid compositions (% dry matter) of *Macrobrachium nipponense,* after a 56-day feeding experiment. Values are expressed as mean ± SD (n = 3). Mean values in the same row with different superscript letters are significantly different (*P* < 0.05).

Fatty acid			Canthaxanthin (mg/kg)			One- way ANOVA		
	Control	50	100	150	200	F	d.f.	P-value (DMRT)
C14:0	3.95 ± 0.02^a^	3.93 ± 0.03^ab^	3.90 ± 0.03^bc^	3.87 ± 0.03^c^	3.80 ± 0.02^d^	18.127	4	0.000
C15:0	0.51 ± 0.01^a^	0.50 ± 0.02^a^	0.46 ± 0.01^b^	0.48 ± 0.02^ab^	0.42 ± 0.02^c^	14.519	4	0.000
C16:0	14.55 ± 0.07^a^	13.54 ± 0.04^b^	12.25 ± 0.02^c^	10.98 ± 0.03^d^	9.49 ± 0.05^e^	591.289	4	0.000
C17:0	0.26 ± 0.02^a^	0.23 ± 0.01^b^	0.23 ± 0.02^b^	0.20 ± 0.02^c^	0.18 ± 0.02^c^	19.072	4	0.000
C18:0	4.36 ± 0.03^d^	4.40 ± 0.02^c^	4.46 ± 0.02^b^	4.53 ± 0.01^a^	4.50 ± 0.02^a^	51.942	4	0.000
C20:0	0.41 ± 0.02^a^	0.37 ± 0.01^b^	0.39 ± 0.02^ab^	0.39 ± 0.01^ab^	0.37 ± 0.01^b^	4.734	4	0.018
C22:0	0.22 ± 0.02^a^	0.19 ± 0.02^bc^	0.21 ± 0.01^ab^	0.18 ± 0.02^cd^	0.16 ± 0.01^d^	8.637	4	0.003
C23:0	0.43 ± 0.02^c^	0.46 ± 0.01^b^	0.42 ± 0.02^c^	0.47 ± 0.02^b^	0.50 ± 0.03^a^	15.189	4	0.000
ΣSFA	24.69 ± 0.06^a^	23.62 ± 0.05^b^	22.32 ± 0.07^c^	21.10 ± 0.04^d^	19.42 ± 0.05^e^	830.426	4	0.000
C16:n	9.73 ± 0.03^e^	10.06 ± 0.07^d^	10.20 ± 0.02^c^	10.35 ± 0.05^b^	10.51 ± 0.06^a^	117.875	4	0.000
C17:n	0.37 ± 0.01^c^	0.39 ± 0.02^bc^	0.42 ± 0.01^ab^	0.45 ± 0.02^a^	0.44 ± 0.02^a^	11.904	4	0.001
C18:n9	33.90 ± 0.10^d^	34.02 ± 0.13^d^	34.69 ± 0.08^c^	35.17 ± 0.06^b^	35.81 ± 0.16^a^	181.438	4	0.000
C20:n9	0.98 ± 0.02^d^	1.02 ± 0.02^c^	1.09 ± 0.01^b^	1.07 ± 0.01^b^	1.22 ± 0.02^a^	163.277	4	0.000
C22:n9	0.19 ± 0.01^d^	0.23 ± 0.02^c^	0.23 ± 0.02^c^	0.25 ± 0.01^b^	0.27 ± 0.01^a^	24.990	4	0.000
ΣMUFA	45.17 ± 0.14^e^	45.72 ± 0.12^d^	46.63 ± 0.08^c^	47.29 ± 0.21^b^	48.25 ± 0.06^a^	291.211	4	0.000
C18:2n6	20.29 ± 0.03^d^	20.38 ± 0.02^cd^	20.44 ± 0.05^bc^	20.53 ± 0.012^b^	20.82 ± 0.09^a^	31.368	4	0.000
C18:3n6	0.55 ± 0.02^e^	0.62 ± 0.01^d^	0.65 ± 0.02^c^	0.74 ± 0.01^b^	0.82 ± 0.01^a^	179.885	4	0.000
C18:3n3	1.71 ± 0.01^d^	1.74 ± 0.01^c^	1.78 ± 0.01^b^	1.85 ± 0.01^a^	1.86 ± 0.01^a^	133.612	4	0.000
C20:2n	2.39 ± 0.02^d^	2.45 ± 0.01^c^	2.47 ± 0.02^c^	2.54 ± 0.02^b^	2.60 ± 0.03^a^	69.477	4	0.000
C20:3n3	0.59 ± 0.01^c^	0.60 ± 0.01^c^	0.64 ± 0.01^b^	0.66 ± 0.01^a^	0.65 ± 0.01^ab^	30.316	4	0.000
C20:5n3(EPA)	2.28 ± 0.01^e^	2.38 ± 0.01^d^	2.42 ± 0.01^c^	2.50 ± 0.01^b^	2.63 ± 0.01^a^	532.676	4	0.000
C22:5n3(DPA)	0.30 ± 0.01^d^	0.33 ± 0.01^c^	0.38 ± 0.01^b^	0.43 ± 0.01^ab^	0.46 ± 0.01^a^	30.749	4	0.000
C22:6n3(DHA)	2.01 ± 0.03^e^	2.14 ± 0.02^d^	2.25 ± 0.03^c^	2.34 ± 0.01^b^	2.47 ± 0.04^a^	180.229	4	0.000
ΣPUFA	30.12 ± 0.15^e^	30.64 ± 0.10^d^	31.03 ± 0.06^c^	31.59 ± 0.12^b^	32.31 ± 0.08^a^	241.177	4	0.000

Abbreviations: SFA: Saturated fatty acid, MUFA: Monounsaturated fatty acid, EPA: Eicosapentaenoic acid, DPA: Docosapentaenoic acid, DHA: Docosahexaenoic acid, PUFA: Polyunsaturated fatty acid.

### Expression of genes associated with growth, immunity, and metabolism

**Fig. 1:**
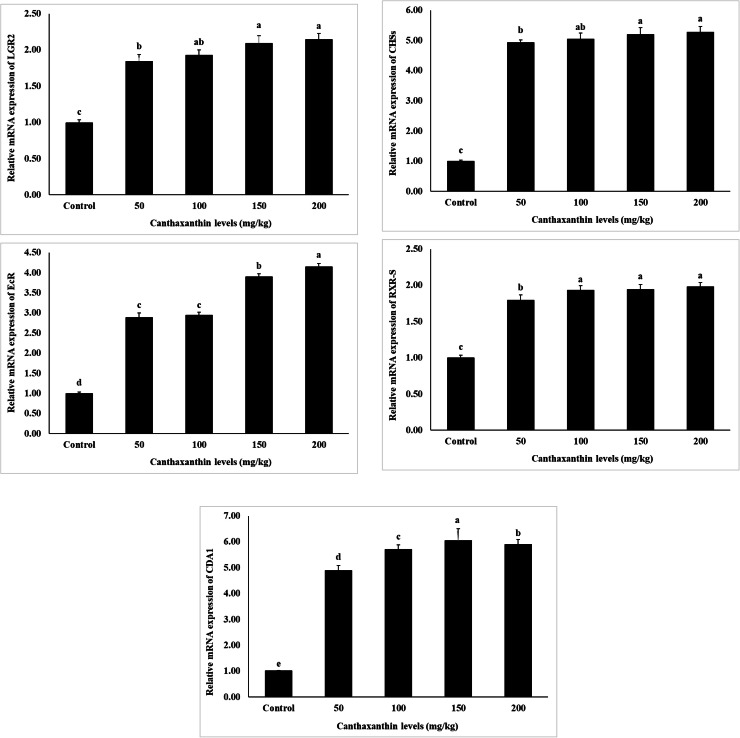
Effects of different dietary canthaxanthin levels on growth-related gene expression of *Macrobrachium nipponense,* after a 56-day feeding experiment. Values are expressed as mean ± SD (n = 3). Mean values in bars with different superscript letters are significantly different (*P* < 0.05).

**Fig. 2:**
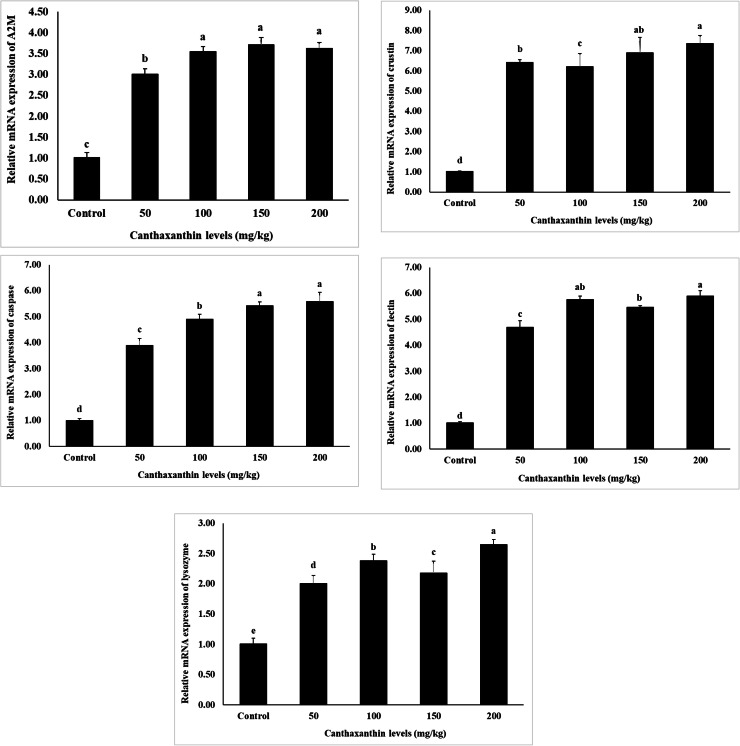
Effects of different dietary canthaxanthin levels on immune-related gene expression of *Macrobrachium nipponense,* after a 56-day feeding experiment. Values are expressed as mean ± SD (n = 3). Mean values in bars with different superscript letters are significantly different (*P* < 0.05).

**Fig. 3:**
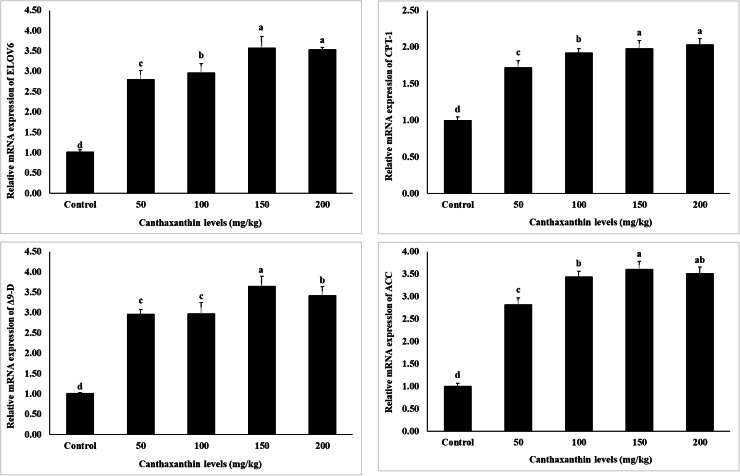
Effects of different dietary canthaxanthin levels on metabolism-related gene expression of *Macrobrachium nipponense,* after a 56-day feeding experiment. Values are expressed as mean ± SD (n = 3). Mean values in bars with different superscript letters are significantly different (*P* < 0.05).

The expression of genes related to growth, immunity, and metabolism is shown in Fig. 1, Fig. 2, Fig. 3. The results indicate that the expression levels of all examined genes, including *alpha-2-macroglobulin (A2* *M), acetyl-CoA carboxylase (ACC), elongation of very long chain fatty acids protein 6 (ELOV6), carnitine O-palmitoyltransferase 1 (CPT-1), delta-9 desaturase (Δ9-D), crustin, cytidine deaminase 1 (CDA1), leucine-rich repeat-containing* G*-protein-coupled receptor 2 (LGR2), ecdysteroid receptor (EcR), lysozyme, retinoid X receptor (RXR-S), lectin, caspase, and chitin synthase (CHSs)*, were significantly influenced by dietary canthaxanthin concentrations (*P* < 0.05). Increased canthaxanthin levels correlated with higher mRNA expression across treatments, with 150 and 200 mg/kg showing the highest levels. The control group had the lowest expression of these genes (*P* < 0.05).

## Discussion

### Water quality indicators

The investigation assessed water quality indicators and found no significant variations in temperature, total water hardness, phosphate, nitrite, ammonium, pH, and nitrate across the canthaxanthin treatment groups. However, dissolved oxygen levels were influenced by canthaxanthin concentrations, with the control group showing the lowest levels and the highest in treatments with 150 and 200 mg/kg of the pigment. These results align with Niu et al. [35], which found that higher astaxanthin concentrations significantly increased dissolved oxygen levels in whiteleg shrimp (*L. vannamei*). The elevated dissolved oxygen levels observed in the canthaxanthin-treated groups, compared to the control, underscore the substantial role of this pigment in mitigating oxidative stress in aquatic organisms. Inadequate nutrition and adverse environmental conditions can disrupt metabolism and increase free radical production. Immune cells are particularly susceptible to oxidative stress due to their reliance on membrane receptors for communication. Incorporating antioxidants, such as carotenoid pigments, into crustacean diets can mitigate the effects of free radicals, enhance organism health, reduce stress, lower oxygen consumption, and indirectly boost dissolved oxygen levels in aquatic environments [7,34,35,56].

### Growth parameters

The results of our research indicate that the inclusion of dietary canthaxanthin significantly improves growth parameters in comparison to the control treatment. Furthermore, the FCR for treatments including canthaxanthin exhibited a marked decrease. These results are consistent with prior research investigating the effects of carotenoid pigments on similar growth metrics across various crustacean species. Notable studies include those by Shen et al. [57] and Fawzy et al. [8], which focused on whiteleg shrimp (*L. vannamei*), Zhang et al. [20] on red swamp crayfish (*Procambarus clarkii*), and Deng et al. [58] on swimming crab (*Portunus trituberculatus*). Additionally, Wang et al. [43] and Wang et al. [59] examined kuruma shrimp (*Marsupenaeus japonicus*) and giant tiger shrimp (*Penaeus monodon*), respectively. A comparative analysis of findings from parallel studies, in conjunction with our research, suggests that increased dietary consumption of carotenoids, particularly canthaxanthin, positively affects growth metrics. This enhancement can be attributed to the significant function that these carotenoid pigments serve as mediators in metabolic processes, which subsequently improves food efficiency by expediting digestion. Additionally, carotenoids are instrumental in lowering energy expenditure by decreasing the duration of the molting cycle process in crustaceans and inhibiting the activity of NADPH, thereby promoting optimal growth in these aquatics. Also, research shows that carotenoids significantly enhance the structural characteristics of the intestine by increasing the width and height of villi, which improves the absorption surface area. Elongated villi are indicative of optimal intestinal function and enhanced nutrient absorption, contributing to improved growth. Carotenoids have been shown to increase the levels of ecdysteroid hormones in the hemolymph of prawns, thereby facilitating growth throughout the molting cycle [[60], [61], [62]]. In the present study, the growth parameters observed in treatments supplemented with a ration containing 200 mg/kg of canthaxanthin pigment were found to be lower than those in the 150 mg/kg treatment group. This decline can be ascribed to the inhibitory effects of carotenoids when administered at excessive levels. Despite the antioxidant properties and significant growth-promoting potential of this pigment, its application beyond the optimal level for any aquatic species may impart a bitter taste to the diet. Consequently, this alteration in palatability can lead to a reduction in food consumption among aquatic animals, ultimately resulting in diminished growth indicators [22,[60], [61], [62]]. The results of our study, along with those of previously cited research, indicate that the observed improvements in growth performance and a reduction in FCR underscore the important and advantageous role of carotenoids in the regulation of energy metabolism and the enhancement of nutrient absorption. These factors have collectively led to superior growth and nutritional outcomes. Therefore, it is reasonable to infer that canthaxanthin, as a carotenoid pigment, has played a significant role in the improved growth and nutritional efficiency noted in the experimental groups that included this pigment, owing to its previously mentioned properties.

### Biochemical indices

The biochemical indices of hemolymph assessed in our study indicate that an increase in dietary supplementation of canthaxanthin is associated with a notable reduction in the levels of creatinine, glucose, urea, triglycerides, and uric acid when compared to the non-supplemented treatment. Conversely, the levels of HDL and LDL demonstrated a marked elevation relative to the control group. It is noteworthy that the concentrations of phosphorus, cholesterol, and calcium exhibited no significant changes in response to varying levels of canthaxanthin, as these parameters are predominantly determined by dietary factors. Furthermore, the experimental treatments involving canthaxanthin supplementation demonstrated a significant reduction in glucose and triglyceride levels in comparison to the group that did not receive this pigment. Glucose serves as a key physiological marker for short-term stress, with levels rising significantly under stress conditions. Studies show that carotenoid-rich diets possess anti-hyperglycemic properties, potentially modulating carbohydrate metabolism via the hepatopancreas and promoting insulin-like peptide (ILPs) synthesis. In prawns treated with canthaxanthin at concentrations of up to 150 mg/kg, reduced creatinine levels suggest improved muscle metabolism and enhanced excretory organ (antennal gland) function [4,63,64]. The absence of significant differences in cholesterol levels across treatments in our study may be attributed to low-stress rearing conditions and adequate nutrition, both of which are vital for lipid metabolism and the prevention of excess hemolymph cholesterol accumulation. Furthermore, the decrease in triglyceride levels in prawns receiving canthaxanthin likely indicates its role in strengthening defense mechanisms against oxidative stress, stemming from carotenoid interactions with fatty acids (esterification) and reduced triglyceride accumulation [64,65].

### Immune responses

The inclusion of dietary canthaxanthin resulted in a substantial elevation in the levels of SGC, THC, TP, GC, HC, and ALB in prawns. In contrast, CORT levels exhibited a significant reduction when compared to the non-supplemented treatment. The results of our study align with prior research concerning the effects of astaxanthin on the aforementioned parameters, as demonstrated in studies conducted by Cheng and Wu [1] on *P. clarkii*, Eldessouki et al. [66], and Chuchird et al. [67] on *L. vannamei*, Flores et al. [68] on postlarvae *L. vannamei*, Weilong et al. [44] and Wang et al. [43] on M*. japonicus*. The findings derived from the aforementioned research underscore the crucial function of carotenoid pigments in bolstering the non-specific immune system and increasing tolerance to environmental pathogens, including bacteria, while also enhancing resilience to environmental stressors. Typically, an increase in THC values in crustaceans is regarded as a marker of disease resistance. Additionally, carotenoids, including canthaxanthin, exert anti-inflammatory effects and positively influence the neuroendocrine-immune system (such as the neuro-secretory gland of the X organ) in prawns, thereby reducing the impacts of stressors and lowering CORT levels in the hemolymph [1,23,66,67]. In this research, it was observed that the levels of the LYZ and PO in groups supplemented with canthaxanthin were notably elevated compared to those without such supplementation. The aforementioned results are consistent with the findings presented by Cheng and Wu [1], which examined the impact of astaxanthin on the activity of the LYZ enzyme in *P. clarkii*. Commonly, PO and LYZ enzymes are key indicators of innate immunity in crustaceans, reflecting overall health and immune functionality. The observed increase in ALB, TP, LYZ, and PO in the experimental groups administered canthaxanthin in this study, along with the consistency of these findings with previous research, underscores the role of carotenoids in the immunomodulation of hepatopancreatic cells. This modulation appears to enhance the anabolic capabilities of these cells, facilitating the synthesis of hemolymph proteins. Notably, PO and LYZ enzymes are critical indicators of innate immunity in crustaceans, as they are instrumental in the eradication of bacteria, particularly Gram-positive strains. Consequently, the activity levels of these enzymes serve as indicators of the overall health and immune functionality of crustaceans [1,23,55,66]. Our results demonstrate a significant decrease in the activities of LDH, AST, AKP, and ALT when compared to the control group. These outcomes align with the findings reported by Chen et al. [52] and Wang et al. [59] on *P. monodon*, also Zhang et al. [20] on *P. clarkii*. The enzymes AST and ALT act as critical amino acid transporters and are integral to protein metabolic pathways; therefore, variations in their activity levels can serve as indicators of the physiological condition of hepatopancreatic cells. Generally, previously mentioned enzymes are found in low concentrations; however, in cases of significant cellular damage, their levels can increase in the hemolymph [1,57,69]. A comparison of our research results with previous studies reveals that canthaxanthin, like other carotenoids, significantly improves the condition of the hepatopancreas in the examined prawn species following treatment.

### Antioxidant status

Recent research findings indicate that elevated dietary levels of canthaxanthin are associated with a significant increase in T-AOC in comparison to the group that did not receive any canthaxanthin supplementation. This observation is connected to previous studies examining the influence of varying levels of carotenoids on juvenile Chinese mitten crab (*Eriocheir sinensis*), juvenile *L. vannamei*, and *P. monodon*, for instance, those conducted by Jiang et al. [3], Fang et al. [22], Niu et al. [24], Chien et al. [70], and Pan et al. [71], respectively. In general, T-AOC serves as an indicator of both enzymatic and non-enzymatic antioxidant activities, thereby reflecting the organism's responses to a range of environmental factors. Variations in antioxidant levels may be observed, and an elevation in T-AOC is indicative of enhanced resistance to oxidative stress in aquatic organisms [22,23,58,72]. Our findings, compared to similar research, show that carotenoid treatments significantly enhance T-AOC, highlighting their antioxidant efficacy. The results of our work demonstrated that an increase in dietary canthaxanthin was associated with a notable decrease in the levels of MDA, CAT, and SOD. These findings are consistent with research conducted by Zhang et al. [41], which examined these indices in *L. vannamei* and reported a notable decrease in SOD and CAT enzyme activities with higher dietary astaxanthin levels contrasted to the control group. Furthermore, studies conducted by Chen et al. [52], Wang et al. [59], and Niu et al. [24] on *P. monodon*, as well as Cheng and Wu [1] on *P. clarkii*, and Han et al. [42] on *P. trituberculatus*, have also revealed a significant decrease in MDA values in astaxanthin-fed experimental treatments relative to the non-supplemented group, corroborating the results of the current research. SOD and CAT are essential elements of the primary antioxidant defense system against free radicals, which serve to alleviate the harmful effects associated with these oxidative agents. These enzymes play a crucial role in preventing the formation of free radicals during oxidative reactions and are vital for the protection of various cellular membrane structures [[73], [74], [75]]. The findings of our study, in conjunction with previously cited research, suggest that the observed decrease in SOD levels corresponds with the role of carotenoids, such as canthaxanthin, in scavenging superoxide anion radicals (O^·−2^) based on their redox properties. This indicates that lower SOD levels are utilized to neutralize and eliminate the aforementioned radicals [74,76,77]. Additionally, the significant reduction in CAT levels observed in subjects administered canthaxanthin suggests a diminished requirement for this enzyme. This phenomenon may be attributed to the decreased production of hydrogen peroxide (H_2_O_2_) metabolites, which is associated with the reduced activity of SOD during the process of free radical mitigation facilitated by the carotenoid pigment [[77], [78], [79]]. As previously indicated, an elevation in canthaxanthin concentrations is linked to a notable decrease in MDA levels, supporting the results of multiple studies. These findings imply that carotenoids are instrumental in mitigating lipid peroxidation, a self-sustaining chain reaction instigated by free radicals. Therefore, the incorporation of carotenoids, including canthaxanthin, into dietary practices can reduce lipid peroxidation levels and provide protective benefits against damage induced by free radicals [79,80].

### Digestive enzyme activity

Our study found that enhanced supplementation of canthaxanthin resulted in a notable improvement in the activity of digestive enzymes when compared to the control group. This finding aligns with the feeding trials conducted by Fawzy et al. [21] and Wang et al. [43], which examined the effects of carotenoid supplementation on the enzyme activities of *L. vannamei*, and M*. japonicus*, respectively. The activity of these enzymes is recognized as a crucial indicator of nutritional condition and growth regulation in aquatics, thereby facilitating the preparation of desirable diets for various crustaceans. Research shows that contribute to the preservation of an acidic intestinal pH, thereby fostering the proliferation of beneficial bacterial species such as *Bacillus* and *Lactobacillus*. These microorganisms are known to synthesize a range of extracellular enzymes, including those involved in digestion. The canthaxanthin pigment enhances the activity of these enzymes and improves digestion, absorption, and nutritional efficiency by modulating bacterial communities and promoting balance among intestinal microorganisms [21,81,82]. Therefore, the consistent findings observed across various studies highlight the notable contribution of carotenoids to the enhancement of nutritional processes in aquatics.

### Intestinal microbiota

The present investigation on intestinal microbiota has identified notable variations in LAB and TBC across the various experimental groups. The results obtained align with those previously documented by Chuchird et al. [67], who examined the impact of varying levels of astaxanthin on *L. vannamei*. Research has demonstrated that carotenoids play a significant role in modulating the composition of intestinal bacteria. By sustaining a balanced acidic environment, these pigments inhibit pathogenic bacteria while promoting beneficial species. Specifically, canthaxanthin has been shown to diminish pathogenic strains and enhance populations of *Lactobacillus* [59,67]. The observed increase in LAB and decrease in TBC in treatments containing canthaxanthin support these previous findings. Canthaxanthin may enhance intestinal microflora by creating competitive conditions and preserving an acidic pH, thereby promoting the growth of gram-positive *Lactobacillus*.

### Body components

Recent research has indicated that an increase in dietary canthaxanthin is associated with a notable enhancement in the TCC across different anatomical parts of the studied prawn species. The results of our investigation align with the findings of Jiang et al. [3] on *E. sinensis*, Fawzy et al. [21] on *L. vannamei*, and Wade et al. [81] on *P. monodon*. Additionally, both crude protein and crude lipid concentrations demonstrated a significant increase in response to higher levels of dietary canthaxanthin. A comprehensive analysis of carcass composition revealed a positive correlation between elevated canthaxanthin supplementation in dietary regimes and improvements in carcass quality. This study supports the conclusions drawn by Jiang et al. [3] regarding *E. sinensis*, Göçer et al. [83] concerning the grooved tiger shrimp (*Penaeus semisulcatus*), as well as those of Wang et al. [59] and Niu et al. [24] related to *P. monodon*. The substantial impact of carotenoids on the metabolism of proteins and lipids offers a plausible explanation for the significant effects of this pigment on the biochemical composition of the experimental prawns [3,59,83]. The observed improvement in whole-body biochemical composition in our investigation, in conjunction with comparable results documented in the previously mentioned studies, can likely be ascribed to the advantageous properties of canthaxanthin, a carotenoid pigment. Canthaxanthin seems to be instrumental in reducing the deterioration of lipids and proteins induced by free radicals generated from metabolic processes and other stressors, which have been demonstrated to increase their concentrations within the carcass. Recent empirical evidence suggests that an increase in dietary canthaxanthin correlates with a significant rise in EAA, MUFA, and PUFA. In contrast, a marked reduction in SFA was observed in groups that received canthaxanthin supplementation. These findings are consistent with the research conducted by Chen et al. [52] on *P. monodon*. The advantageous effects of carotenoids in enhancing levels of EAA and PUFA may be attributed to their antioxidant characteristics, which protect these compounds from oxidative degradation. Furthermore, the noted decrease in SFA associated with carotenoid pigments may be a consequence of the stimulation of desaturation and elongation processes, thereby contributing to the observed increase in PUFA levels. However, the specific mechanisms through which carotenoid pigments affect amino acid and fatty acid metabolism warrant further investigation [52,84].

### Expression of genes associated with growth, immunity, and metabolic processes

Our research indicates that increased supplementation of canthaxanthin significantly enhances the expression of genes associated with growth, immune function, and metabolic processes. This finding aligns with the feeding trials conducted by Zhang et al. [41] and Niu et al. [24], which examined the effects of astaxanthin on gene expression in *P. monodon* and *L. vannamei*, respectively. Previous research has established that carotenoids enhance the expression of toll-like receptors (TLRs), which are essential for activating the innate immune response in crustaceans. These receptors operate by identifying specific molecular patterns and relaying signals to pattern recognition receptors (PRRs). This cascade of events, triggered by immune system activation in crustaceans, ultimately results in the upregulation of various genes [24,41,51].

## Conclusion

The results of our study demonstrate that increased supplementation of canthaxanthin markedly improves growth performance, hemato-biochemical indices, immunological responses, and metabolic changes in the oriental river prawn. This underscores the critical role of canthaxanthin as an important carotenoid in promoting growth metrics, enhancing immune system functionality, and improving digestibility. Based on these findings, it is recommended to incorporate a dietary supplementation of 150 mg/kg of canthaxanthin to optimize the aforementioned parameters in this freshwater species.

## CRediT authorship contribution statement

**Mohammad Ettefaghdoost:** Writing – review & editing, Writing – original draft, Visualization, Supervision, Project administration, Methodology, Investigation, Funding acquisition, Formal analysis, Conceptualization. **Hamid Navirian:** Validation, Resources, Investigation, Data curation, Conceptualization. **Hossein Haghighi:** Writing – original draft, Validation, Software, Methodology, Investigation, Formal analysis.

## Declaration of competing interest

The authors declare that they have no known competing financial interests or personal relationships that could have appeared to influence the work reported in this paper.

## Data Availability

Data will be made available on request.
